# A longitudinal evaluation of the community-based rehabilitation support programme for Post-COVID-19 condition in Hong Kong

**DOI:** 10.1038/s41598-026-46888-x

**Published:** 2026-03-31

**Authors:** Leonard Ho, Ka Wai Yuen, Ming Hong Kwong, Sam Ho Sum Yuen, Pui Chee Hung, Dexing Zhang, Samuel Yeung Shan Wong, Vincent Chi Ho Chung

**Affiliations:** Jockey Club School of Public Health and Primary Care, Faculty of Medicine, The Chinese University of Hong Kong, Shatin, New Territories, Hong Kong

**Keywords:** COVID-19, Rehabilitation, Community health services, Integrated care, Patient reported outcome measures, Health care, Public health

## Abstract

Eight non-governmental organisations (NGOs) in Hong Kong participated in a one-year pilot programme delivering community-based multidisciplinary rehabilitation services for individuals with Post-COVID-19 Condition (PCC). This study evaluated the programme’s performance across three NGOs, focusing on improvements in patient-reported outcomes and recovery trajectories, and exploring associations with sociodemographic characteristics and service utilisation. A prospective longitudinal survey was conducted between November 2022 and July 2023. Health outcomes were assessed using the Modified COVID-19 Yorkshire Rehabilitation Scale (C19-YRSm) at baseline, immediately post-service, and three months post-service. Sociodemographic and service utilisation data were also collected. Univariate and multivariate logistic regression analyses were performed to identify the potential predictors for significant clinical improvements. Of the 1,655 recruited participants, 623 completed all three assessments. Statistically significant improvements were observed in 16 out of 17 outcome measures regarding symptom severity, functional disability, and overall health scores. Despite these improvements, participants did not fully return to their self-reported pre-COVID-19 health status. Regression analyses revealed that younger participants and those with more severe symptoms prior to service were more likely to exhibit substantial clinical improvements across multiple domains immediately and three months after service utilisation. This community-based PCC multidisciplinary rehabilitation programme in Hong Kong yielded significant improvements in health outcomes, though complete recovery was not achieved. Despite the lack of a control group, our findings underscored the potential effectiveness of person-centred, case-managed, and multidisciplinary rehabilitation packages.

**Supplementary Information:**

The online version contains supplementary material available at 10.1038/s41598-026-46888-x.

## Introduction

Post-COVID-19 Condition (PCC), also known as “Long COVID-19”, is the continuation or development of new symptoms three months following the initial SARS-CoV-2 infection, and those symptoms last for at least two months without other explanations^1^. PCC is heterogenous and multi-systemic which means that an individual with this condition can present with a wide variety of symptoms including, but not limited to, fatigue, shortness of breath, muscle pain, joint pain, headache, cough, chest pain, altered smell and taste, and diarrhoea^2–4^. Cognitive impairment, memory loss, and sleep disorders are also not uncommon^2–4^.

A meta-analysis revealed that around 45% of people recovered from COVID-19 would experience at least one unresolved symptom for more than four months^5^, while a large-scale retrospective cohort study revealed that PCC symptoms may persist for more than two years^6^. Meanwhile, in the United Kingdom, the Office of National Statistics estimated that around 1.5 million people experiencing self-reported PCC reported that the symptoms adversely affected their day-to-day activities^7^. About one-fifth of them reported that they could not live alone without assistance and either had reduced ability to work or had to leave work altogether^7^. Another review found that over half of the individuals with PCC would have a significantly poor quality of life attributable to persistent fatigue, dyspnoea, anosmia, sleep disturbances, and mental health issues^4^. Given the prevalence of PCC and its impacts on personal lives and countries’ productivity, the World Health Organization have developed living guidelines for health and social care providers on the design and implementation of rehabilitation services^8^. The document emphasises the importance of multidisciplinary rehabilitation teams, continuity and coordination of care, and people-centred care and shared decision-making and expects the providers to have in place standardised symptoms assessment and outcome measurement tools, follow-up system, and referral system^8^.

Responding to the emergence of PCC cases in Hong Kong, where 51.9% of individuals with prior COVID-19 infection were reported to develop PCC in 2023^9^, eight non-governmental organisations (NGOs) participated in a pilot programme to support individuals by co-developing personalised rehabilitation plans with service users and facilitating referrals to appropriate health and allied health services delivered within community settings, leveraging existing local primary care networks^10^. In this longitudinal study, we aimed to (i) evaluate the performance of this community-based rehabilitation support programme on patient-reported outcomes and recovery trajectories and (ii) examine the associations between sociodemographic and service utilisations and recovery progress.

## Methods

### Outline of the community rehabilitation support programme

This one-year programme, launched in September 2022, involved eight NGOs to support individuals with self-reported rehabilitation needs, prioritising vulnerable groups such as older adults living in residential care homes and those requiring care. The NGOs were responsible for assessing eligibility, assigning case managers (social workers), and co-developing personalised rehabilitation plans of up to 12 weeks with service users. Referrals to appropriate community-based health and allied health services were made in due course, with the agreement of all parties involved. All services were personalised, provided free of charge, and delivered by multidisciplinary teams comprising traditional Chinese medicine practitioners (TCMPs), biomedically trained doctors (BMDs), physiotherapists, occupational therapists, nurses, and dietitians. Services were offered either in close proximity to service users’ residences or at participating NGOs. Specifically, BMDs and nurses delivered a range of conventional medical services, including symptom management and general health advice and education; TCMPs provided Chinese herbal medicine, acupuncture, and Chinese massage; and allied health professionals conducted exercise sessions and nutritional consultations. Case managers regularly reviewed participants’ rehabilitation progress in consultation with both service users and providers, making adjustments to care plans as needed.

During September 2022 to March 2023, a total of 10,000 eligible individuals was accepted on a first-come, first-served basis, meeting the following criteria:

(i) Confirmed COVID-19 recovery, evidenced by a positive test through Hong Kong Government-recognised testing bodies via RT-PCR (reverse transcription-polymerase chain reaction) or declaration through the Hong Kong Department of Health’s *Declaration System for Individuals Tested Positive for COVID-19 Using Rapid Antigen Test*;
(ii) Experiencing PCC, defined as symptoms appearing within three months of COVID-19 onset and persisting for at least two months;^1^ and.
(iii) No prior participation in the *Special Chinese Medicine Out-patient Programme for Discharged COVID-19 Patients* offered by the Hong Kong Hospital Authority.

The NGOs had full discretion to develop the most suitable rehabilitation plans with participants and monitor their progress, without the requirement to disclose specific details of service arrangements and utilisation due to privacy concerns.

### Study design and participants

From November 2022 to July 2023, we recruited 1,655 users of the community-based programme through three of the eight NGOs to participate in this prospective pre- and post-intervention survey study. Participants were recruited consecutively as they enrolled in the programme and met the eligibility criteria mentioned below. These three NGOs offer a wide range of services, including community health and social care, health education, and residential care, targeting the general public as well as individuals with disabilities or chronic conditions. The selected NGOs were chosen to represent a diverse cross-section of service provision and participant characteristics.

The inclusion criteria of this programme included: (i) being a service user of one of the NGOs; (ii) being aged 18 or above; (iii) being proficient in both written and oral Cantonese; and (iv) being capable of providing informed consent. Individuals with conditions (e.g., serious cognitive impairment) that impeded their participation or adherence were excluded. The study complied with the Declaration of Helsinki and was approved by the Joint Chinese University of Hong Kong–New Territories East Cluster Clinical Research Ethics Committee (Reference number: 2022.268). All participants were informed of the study details and gave consent prior to data collection. An incentive of HKD300 cash coupons was offered to those who completed all required questionnaires.

### Outcome assessment and data collection

We evaluated participants’ outcomes using the Modified COVID-19 Yorkshire Rehabilitation Scale (C19‐YRSm), a patient‐reported outcome instrument developed to assess the symptom severity, functional disability, and overall health state experienced by individuals with PCC^11^. The severity of each of the ten symptoms was rated by the participant on a four-point Likert scale, with 0 being the absence of the symptom and 3 being the presence of the symptom in a life-disturbing degree. A symptom might have more than one item describing its details. For instance, the symptom of cognition covered problems with concentration, problems with memory, and problems with planning. For those symptoms with multiple items, their severity score referred to the worst score among all items. The degree of disability across five functional areas was also rated on a four-point Likert scale. The overall health scores before COVID-19 and the present were rated from 0 to 10, with 0 being the worst health the participant could imagine and 10 being the best health the participant could imagine. Moreover, 25 predefined symptoms were recorded in terms of their presence during the last seven days.

We obtained authorisation from the original C19-YRSm developers to formally translate the instrument from English to Hong Kong Cantonese, using the five-step forward–backward translation method^12^. Before the survey, we evaluated the content validity of the Cantonese translation in individual cognitive debriefing sessions for individuals with PCC (*n* = 10). The data collection took place at three specific follow-up timepoints: (i) before service utilisation (baseline); (ii) immediately after service utilisation (three months from baseline); and (iii) three months after service utilisation (six months from baseline). Data were collected from participants through telephone interviews, online questionnaires, or both. Online questionnaires were administered via the Qualtrics platform (Qualtrics, Provo, Utah, United States). Participants’ demographical and clinical characteristics were also assessed before service utilisation.

### Data analysis

We performed data analysis using Python 3.12 (Python Software Foundation, Wilmington, Delaware, United States) with *SciPy*^13^ and *statsmodels*^14^, and created graphical representations using Microsoft Excel 2016 (Microsoft Corporation, Redmond, Washington, United States). Continuous data were represented as mean and standard deviation (SD), while categorical data were expressed as frequencies and percentages.

Wilcoxon Signed Ranks Test was used to compare symptom severity scores, functional disability scores, and overall health scores between pre-COVID-19 infection and three months after service utilisation. These pairwise comparisons were pre-specified to focus on the baseline-to-three-month interval, and no additional post-hoc comparisons between intermediate time points were planned. Friedman test was performed to compare the changes in symptom severity scores and functional disability scores, the number of the 25 predefined symptoms reported by the participants, and the overall health scores across the three timepoints. A symptom was defined as being present in a patient if the severity score was greater than 0. For the comparison of the presence of symptoms and functional disabilities, Cochrane’s Q test was used to evaluate differences across the three timepoints, with McNemar’s test used for comparisons between baseline and three months, consistent with the pre-specified focus on this interval. All statistical tests were two-sided, with statistical significance established at *p* < 0.05; no formal adjustment for multiple comparisons was applied, as the primary analyses were limited to the pre-specified baseline-to-three-month interval.

We then performed logistic regression analyses to explore the associations between the achievement of minimally important differences (MIDs) three months after service utilisation and demographic and clinical characteristics. The dependent variables were defined as achieving an MID (i.e., improvement greater than one-half of the baseline SD)^15^ in symptom severity scores, functional disability scores, number of other symptoms, and overall health scores. There was a total of 14 independent variables, including:

(i) Age;
(ii) Female (biological sex);
(iii) Body mass index;
(iv) Smoking;
(v) Full-time employment before COVID-19;
(vi) Number of COVID-19 infection(s);
(vii) Number of COVID-19 vaccine dose(s);
(viii) Number of chronic disease(s), as reported by participants to their NGO;
(ix) Stroke history;
(x) Symptom severity scores, functional disability scores, number of other symptoms, or overall health scores before infection;
(xi) Symptom severity scores, functional disability scores, number of other symptoms, or overall health scores before service utilisation;
(xii) Usage of conventional medicine services (provided by BMDs);
(xiii) Usage of traditional Chinese medicine (TCM) services; and.
(xiv) Usage of allied health services.

Body mass index was included as overweight and obesity have been associated with prolonged PCC symptoms^16^. Smoking was considered because it may increase the risk of PCC^17^. Pre-infection psychological distress has also been linked to PCC symptom severity;^18^ however, as it could not be measured retrospectively in our survey, pre-infection full-time employment was used as a proxy indicator. Stroke history was included as stroke survivors are more prone to long-term cognitive decline^19^.

Univariate logistic regression analyses were initially performed to identify independent variables with statistically significant effects on the dependent variables. Variables demonstrating a *p*-value smaller than 0.1 were subsequently qualified for multivariate logistic regression analyses to assess their predictability for the dependent variables while controlling for potential confounders. A more liberal cutoff of 0.1 was chosen to focus on identifying potential predictor variables rather than to test a hypothesis^20^. We presented the results from the univariate and multivariate logistic regression analyses using odds ratios (ORs) and adjusted odds ratios (aORs), respectively, as well as their corresponding 95% confidence intervals (CIs) and *p*-values.

## Results

### Characteristics of participants

We recruited 1,655 of the 10,000 NGO service users for this study, of whom 1,276 (77%) completed at least one questionnaire. Among these, only 623 (194 males and 429 females) completed all three questionnaires (i.e., before service utilisation, immediately after service utilisation, and three months post-service), resulting in a completion rate of 48.82%. The average age of the participants was 51.96 ± 16.0 years. Seventy-two (11.56%) reported having smoked more than 100 cigarettes in their lifetime, classifying them as regular smokers^21^. A total of 278 participants (44.62%) reported no change in employment or study status following COVID-19 infection, while the remainder experienced changes in their employment. Most participants (79.45%) had only one COVID-19 infection. Vaccination coverage was high, with 435 individuals (69.83%) having received three or more doses. Over half (334; 53.61%) reported no chronic diseases. Until three months after service utilisation, among the three health services provided, TCM services were the most frequently used, with a mean number of usages of 5.79 ± 2.87 times, compared to 0.09 ± 0.35 for conventional medicine services and 1.13 ± 2.85 for allied health services. Table 1 presents other demographic and clinical characteristics of the participants.

**Table 1:** Demographical and clinical characteristics of participants (*N* = 623).

Demographic Characteristics	All participants^*^
**Age (years)**	51.96 ± 16.00
Age group	
≤ 37	140 (22.47%)
37–46	104 (16.69%)
47–56	94 (15.09%)
57–66	145 (23.27%)
≥ 66	140 (22.47%)
**Male**	194 (31.14%)
**Body mass index (kg/m** ^**2**^ **)**	23.33 ± 3.96
Degree of education	
Primary or below	70 (11.24%)
Secondary (Junior, Senior, Sixth form)	300 (48.15%)
Vocational training/Non-degree post-secondary	60 (9.63%)
Undergraduate or above	193 (30.98%)
**Smoke regularly** ^†^	72 (11.56%)
Employment status before COVID-19 infection	
Full-time employed	273 (43.82%)
Part-time employed	74 (11.88%)
Unemployed	22 (3.53%)
Retired	151 (24.24%)
Homemaker	98 (15.73%)
Student	5 (0.80%)
Employment status changed due to COVID-19 (self-reported)	
Stopped working	5 (0.80%)
Lost job	20 (3.21%)
Had to retire/Changed job	84 (13.48%)
On reduced working hours	103 (16.53%)
Other changes in working arrangement	133 (21.35%)
No change	278 (44.62%)
Number of COVID-19 infection	
1	495 (79.45%)
2	124 (19.90%)
3 or more	4 (0.64%)
Number of COVID-19 vaccine doses	
0	33 (5.30%)
1	21 (3.37%)
2	134 (21.51%)
3	341 (54.74%)
≥ 4	94 (15.09%)
Number of chronic disease(s)	
0	334 (53.61%)
1	147 (23.60%)
2	74 (11.88%)
3	43 (6.90%)
≥ 4	25 (4.01%)
**With stroke history**	13 (2.09%)
Utilisation of health services (frequency, until 3 months after service)	
Conventional medicine services	0.09 ± 0.35
Traditional Chinese medicine services	5.79 ± 2.87
Allied health services	1.13 ± 2.85

^*^Data expressed in *n* (percentage) or means ± standard deviation.

^†^Smoke regularly is defined as smoking more than 100 cigarettes during lifetime.

### Symptom and functional assessment

Our pre- and post-intervention results demonstrated statistically significant differences in 16 of the 17 items measured by the C19-YRSm among participants who joined the programme. The only item that did not show a significant difference was personal care, which refers to difficulties with tasks such as using the toilet or getting washed and dressed. The overall health score also improved significantly, rising from 5.85 ± 1.74 before service utilisation to 6.35 ± 1.71 after service utilisation, and remained stable at 6.40 ± 1.81 three months later (*p* < 0.001). Details of the presence and severity of symptoms, functional disabilities, and overall health scores across the three timepoints are shown in Table 2.

**Table 2:** Presence and scores of symptoms, functional disabilities, number of other symptoms, and overall health before service utilisation, after service utilisation, and three months after service utilisation.

	Presence^*^				Score^†^/Number^†^			
	Before service	After service	3 months after service	*P* value	Before service	After service	3 months after service	*P* value
Symptoms								
**Breathlessness**	514 (82.50%)	469 (75.28%)	431 (69.18%)	**< 0.001**	1.38 ± 0.91	1.13 ± 0.87	1.02 ± 0.88	**< 0.001**
At rest	222 (35.63%)	169 (27.13%)	148 (23.76%)	**< 0.001**	0.49 ± 0.74	0.35 ± 0.64	0.30 ± 0.60	**< 0.001**
Changing position	187 (30.02%)	143 (22.95%)	119 (19.10%)	**< 0.001**	0.39 ± 0.67	0.28 ± 0.57	0.23 ± 0.52	**< 0.001**
On dressing	137 (21.99%)	97 (15.57%)	83 (13.32%)	**< 0.001**	0.26 ± 0.54	0.18 ± 0.45	0.17 ± 0.47	**< 0.001**
On walking up stairs	496 (79.61%)	457 (73.35%)	420 (67.42%)	**< 0.001**	1.32 ± 0.92	1.09 ± 0.86	0.98 ± 0.87	**< 0.001**
**Cough/Throat sensitivity/Voice change**	476 (76.40%)	411 (65.97%)	406 (65.17%)	**< 0.001**	1.22 ± 0.91	0.92 ± 0.84	0.95 ± 0.86	**< 0.001**
Cough/Throat sensitivity	456 (73.19%)	398 (63.88%)	386 (61.96%)	**< 0.001**	1.15 ± 0.90	0.88 ± 0.83	0.87 ± 0.83	**< 0.001**
Change of voice	209 (33.55%)	180 (28.89%)	196 (31.46%)	0.094	0.47 ± 0.75	0.38 ± 0.68	0.43 ± 0.72	0.055
**Fatigue**	553 (88.76%)	512 (82.18%)	450 (72.23%)	**< 0.001**	1.60 ± 0.88	1.25 ± 0.85	1.08 ± 0.86	**< 0.001**
**Smell/Taste**	217 (34.83%)	142 (22.79%)	137 (21.99%)	**< 0.001**	0.51 ± 0.80	0.30 ± 0.62	0.30 ± 0.64	**< 0.001**
Altered smell	178 (28.57%)	123 (19.74%)	118 (18.94%)	**< 0.001**	0.41 ± 0.74	0.26 ± 0.56	0.26 ± 0.59	**< 0.001**
Altered taste	177 (28.41%)	112 (17.98%)	105 (16.85%)	**< 0.001**	0.39 ± 0.71	0.23 ± 0.55	0.23 ± 0.56	**< 0.001**
**Pain/Discomfort**	532 (85.39%)	497 (79.78%)	464 (74.48%)	**< 0.001**	1.55 ± 0.94	1.30 ± 0.92	1.18 ± 0.93	**< 0.001**
Chest pain	216 (34.67%)	160 (25.68%)	164 (26.32%)	**< 0.001**	0.47 ± 0.74	0.33 ± 0.63	0.33 ± 0.62	**< 0.001**
Joint pain	393 (63.08%)	349 (56.02%)	355 (56.98%)	**< 0.001**	1.07 ± 1.01	0.89 ± 0.94	0.87 ± 0.92	**< 0.001**
Muscle pain	392 (62.92%)	382 (61.32%)	331 (53.13%)	**< 0.001**	1.02 ± 0.97	0.89 ± 0.86	0.76 ± 0.85	**< 0.001**
Headache	350 (56.18%)	303 (48.64%)	275 (44.14%)	**< 0.001**	0.85 ± 0.91	0.65 ± 0.79	0.59 ± 0.77	**< 0.001**
Abdominal pain	187 (30.02%)	161 (25.84%)	151 (24.24%)	**0.007**	0.41 ± 0.72	0.34 ± 0.64	0.30 ± 0.59	**< 0.001**
**Cognition**	530 (85.07%)	491 (78.81%)	449 (72.07%)	**< 0.001**	1.56 ± 0.95	1.23 ± 0.89	1.09 ± 0.89	**< 0.001**
Concentration	426 (68.38%)	369 (59.23%)	334 (53.61%)	**< 0.001**	1.10 ± 0.94	0.83 ± 0.83	0.71 ± 0.78	**< 0.001**
Memory	519 (83.31%)	455 (73.03%)	419 (67.26%)	**< 0.001**	1.48 ± 0.95	1.12 ± 0.89	1.00 ± 0.90	**< 0.001**
Planning	353 (56.66%)	303 (48.64%)	254 (40.77%)	**< 0.001**	0.90 ± 0.95	0.65 ± 0.80	0.55 ± 0.75	**< 0.001**
**Palpitations/Dizziness**	392 (62.92%)	320 (51.36%)	292 (46.87%)	**< 0.001**	0.95 ± 0.90	0.69 ± 0.79	0.61 ± 0.76	**< 0.001**
Palpitations	291 (46.71%)	224 (35.96%)	208 (33.39%)	**< 0.001**	0.66 ± 0.83	0.47 ± 0.71	0.43 ± 0.69	**< 0.001**
Dizziness	325 (52.17%)	270 (43.34%)	236 (37.88%)	**< 0.001**	0.75 ± 0.87	0.57 ± 0.75	0.50 ± 0.72	**< 0.001**
**Post-exertional malaise**	474 (76.08%)	401 (64.37%)	366 (58.75%)	**< 0.001**	1.30 ± 0.96	0.93 ± 0.86	0.85 ± 0.87	**< 0.001**
**Anxiety/Mood**	386 (61.96%)	357 (57.30%)	326 (52.33%)	**< 0.001**	0.98 ± 0.95	0.81 ± 0.85	0.74 ± 0.85	**< 0.001**
Anxiety	336 (53.93%)	309 (49.60%)	269 (43.18%)	**< 0.001**	0.80 ± 0.89	0.67 ± 0.81	0.60 ± 0.81	**< 0.001**
Depression	266 (42.70%)	236 (37.88%)	205 (32.91%)	**< 0.001**	0.64 ± 0.87	0.50 ± 0.73	0.46 ± 0.75	**< 0.001**
Unwanted memories	180 (28.89%)	164 (26.32%)	145 (23.27%)	**0.011**	0.43 ± 0.77	0.35 ± 0.66	0.32 ± 0.65	**0.001**
Unpleasant dreams	121 (19.42%)	113 (18.14%)	114 (18.30%)	0.729	0.29 ± 0.67	0.26 ± 0.62	0.26 ± 0.60	0.499
Avoid thoughts	131 (21.03%)	118 (18.94%)	118 (18.94%)	0.381	0.30 ± 0.64	0.26 ± 0.59	0.25 ± 0.59	0.122
**Sleep**	501 (80.42%)	440 (70.63%)	433 (69.50%)	**< 0.001**	1.55 ± 1.01	1.15 ± 0.96	1.13 ± 0.97	**< 0.001**
Functional disabilities								
Communication	342 (54.90%)	292 (46.87%)	276 (44.30%)	**< 0.001**	0.83 ± 0.91	0.63 ± 0.78	0.60 ± 0.78	**< 0.001**
Mobility	204 (32.74%)	174 (27.93%)	147 (23.60%)	**< 0.001**	0.48 ± 0.80	0.38 ± 0.68	0.34 ± 0.68	**< 0.001**
Personal care	57 (9.15%)	60 (9.63%)	67 (10.75%)	0.461	0.12 ± 0.42	0.12 ± 0.42	0.14 ± 0.45	0.612
Daily living	231 (37.08%)	185 (29.70%)	165 (26.48%)	**< 0.001**	0.56 ± 0.83	0.39 ± 0.68	0.36 ± 0.68	**< 0.001**
Social role	164 (26.32%)	149 (23.92%)	135 (21.67%)	**0.040**	0.38 ± 0.73	0.31 ± 0.61	0.28 ± 0.59	**0.002**
**Number of other symptoms** ^**‡**^	NA	NA	NA	NA	3.61 ± 4.88	2.75 ± 4.79	2.30 ± 4.82	**< 0.001**
**Overall health** ^**§**^	NA	NA	NA	NA	5.85 ± 1.74	6.35 ± 1.71	6.40 ± 1.81	**< 0.001**

NA: Not applicable.

^*^Data expressed in *n* (percentage).

^†^Data expressed in means ± standard deviation.

^‡^Out of 25 predefined symptoms.

^§^Zero refers to the worst overall health, and 10 refers to the best overall health.

**Fig. 1:**
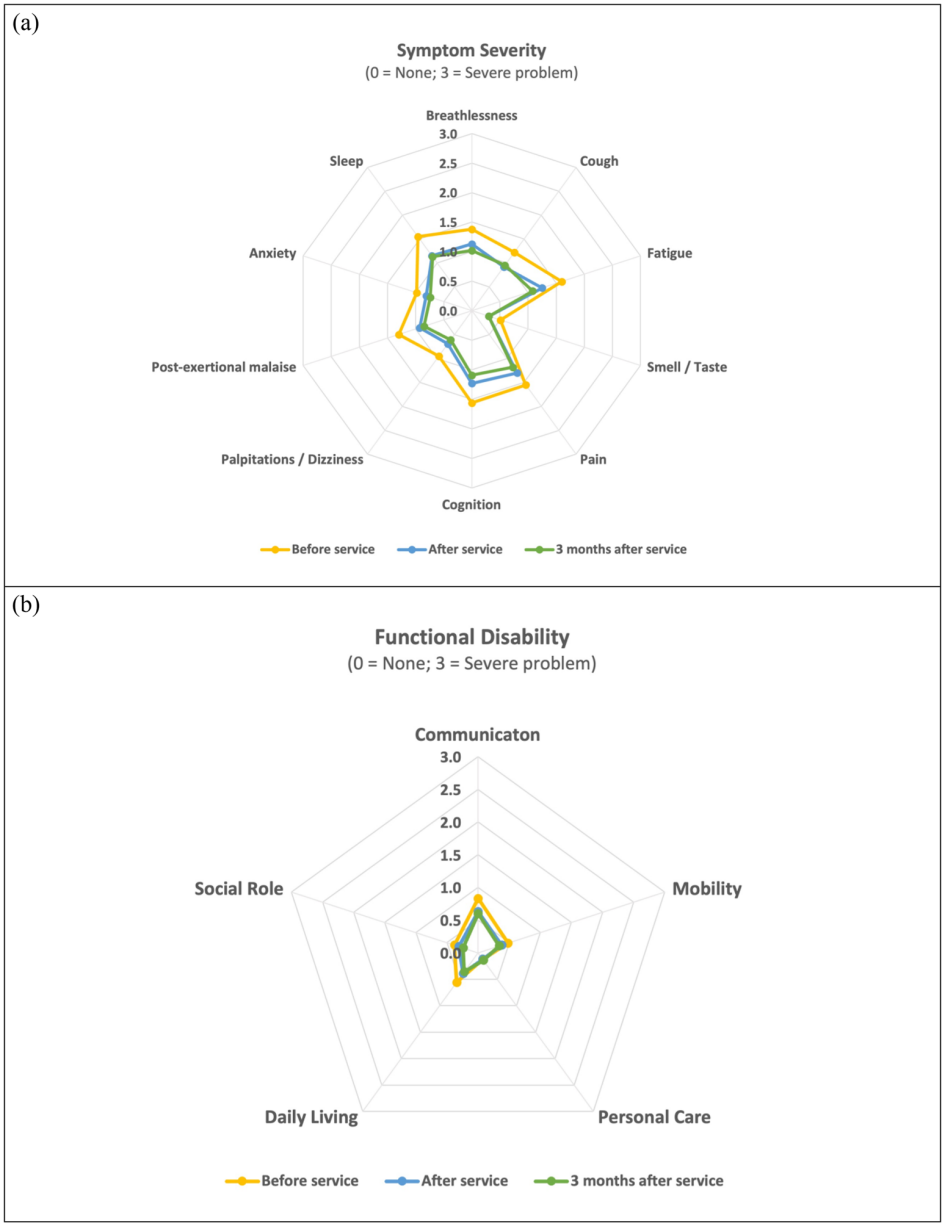
Radar plots of scores of (**a**) symptom severity and (**b**) functional disability before service utilisation, after service utilisation, and three months after service utilisation.

The results were also visualised using radar plots. Figure 1 below presents radar plots depicting the symptom severity and functional disability scores of the participants. These plots illustrate significant differences in both symptoms (Fig. 1a) and functional disabilities (Fig. 1b), as evidenced by the decreased area of the polygons. It is noteworthy that the severity scores and presence of symptoms are generally higher than those of functional disabilities, highlighting the more pronounced impact of symptoms compared to functional impairments.

Even before contracting COVID-19, participants exhibited varying presence and severity across the ten symptoms and five functional disabilities. Table 3 presents the presence and scores for symptoms, functional disabilities, and overall health both prior to COVID-19 infection and three months after service utilisation. In terms of both presence and severity, symptoms, functional disabilities, and overall health remained significantly different (all *p* < 0.001, except for the presence of breathlessness while dressing, where *p* = 0.001), meaning that participants were unable to return to their self-reported pre-COVID-19 health status. The overall health score three months after service (6.40 ± 1.81) was one point lower than before COVID-19 infection (7.47 ± 1.79).

**Table 3:** Presence and scores of symptoms, functional disabilities, number of other symptoms, and overall health before COVID-19 infection and three months after service utilisation.

	Presence^*^			Score^†^/Number^†^		
	Pre-COVID	3 months after service	*P* value	Pre-COVID	3 months after service	*P* value
Symptoms						
**Breathlessness**	301 (48.31%)	431 (69.18%)	**< 0.001**	0.61 ± 0.73	1.02 ± 0.88	**< 0.001**
At rest	75 (12.04%)	148 (23.76%)	**< 0.001**	0.16 ± 0.49	0.30 ± 0.60	**< 0.001**
Changing position	66 (10.59%)	119 (19.10%)	**< 0.001**	0.14 ± 0.46	0.23 ± 0.52	**< 0.001**
On dressing	49 (7.87%)	83 (13.32%)	**0.001**	0.10 ± 0.39	0.17 ± 0.47	**< 0.001**
On walking up stairs	281 (45.10%)	420 (67.42%)	**< 0.001**	0.56 ± 0.71	0.98 ± 0.87	**< 0.001**
**Cough/Throat sensitivity/Voice change**	178 (28.57%)	406 (65.17%)	**< 0.001**	0.39 ± 0.71	0.95 ± 0.86	**< 0.001**
Cough/Throat sensitivity	168 (26.97%)	386 (61.96%)	**< 0.001**	0.36 ± 0.68	0.87 ± 0.83	**< 0.001**
Change of voice	63 (10.11%)	196 (31.46%)	**< 0.001**	0.16 ± 0.52	0.43 ± 0.72	**< 0.001**
**Fatigue**	283 (45.43%)	450 (72.23%)	**< 0.001**	0.59 ± 0.75	1.08 ± 0.86	**< 0.001**
**Smell/Taste**	70 (11.24%)	137 (21.99%)	**< 0.001**	0.18 ± 0.56	0.30 ± 0.64	**< 0.001**
Altered smell	58 (9.31%)	118 (18.94%)	**< 0.001**	0.14 ± 0.50	0.26 ± 0.59	**< 0.001**
Altered taste	51 (8.19%)	105 (16.85%)	**< 0.001**	0.14 ± 0.52	0.23 ± 0.56	**< 0.001**
**Pain/Discomfort**	334 (53.61%)	464 (74.48%)	**< 0.001**	0.69 ± 0.76	1.18 ± 0.93	**< 0.001**
Chest pain	82 (13.16%)	164 (26.32%)	**< 0.001**	0.17 ± 0.47	0.33 ± 0.62	**< 0.001**
Joint pain	231 (37.08%)	355 (56.98%)	**< 0.001**	0.47 ± 0.70	0.87 ± 0.92	**< 0.001**
Muscle pain	198 (31.78%)	331 (53.13%)	**< 0.001**	0.40 ± 0.67	0.76 ± 0.85	**< 0.001**
Headache	155 (24.88%)	275 (44.14%)	**< 0.001**	0.32 ± 0.62	0.59 ± 0.77	**< 0.001**
Abdominal pain	80 (12.84%)	151 (24.24%)	**< 0.001**	0.16 ± 0.47	0.30 ± 0.59	**< 0.001**
**Cognition**	277 (44.46%)	449 (72.07%)	**< 0.001**	0.54 ± 0.69	1.09 ± 0.89	**< 0.001**
Concentration	168 (26.97%)	334 (53.61%)	**< 0.001**	0.33 ± 0.60	0.71 ± 0.78	**< 0.001**
Memory	236 (37.88%)	419 (67.26%)	**< 0.001**	0.46 ± 0.67	1.00 ± 0.90	**< 0.001**
Planning	128 (20.55%)	254 (40.77%)	**< 0.001**	0.26 ± 0.55	0.55 ± 0.75	**< 0.001**
**Palpitations/Dizziness**	159 (25.52%)	292 (46.87%)	**< 0.001**	0.31 ± 0.59	0.61 ± 0.76	**< 0.001**
Palpitations	113 (18.14%)	208 (33.39%)	**< 0.001**	0.22 ± 0.52	0.43 ± 0.69	**< 0.001**
Dizziness	117 (18.78%)	236 (37.88%)	**< 0.001**	0.23 ± 0.52	0.50 ± 0.72	**< 0.001**
**Post-exertional malaise**	218 (34.99%)	366 (58.75%)	**< 0.001**	0.42 ± 0.65	0.85 ± 0.87	**< 0.001**
**Anxiety/Mood**	205 (32.91%)	326 (52.33%)	**< 0.001**	0.41 ± 0.66	0.74 ± 0.85	**< 0.001**
Anxiety	157 (25.20%)	269 (43.18%)	**< 0.001**	0.30 ± 0.58	0.60 ± 0.81	**< 0.001**
Depression	128 (20.55%)	205 (32.91%)	**< 0.001**	0.25 ± 0.55	0.46 ± 0.75	**< 0.001**
Unwanted memories	67 (10.75%)	145 (23.27%)	**< 0.001**	0.14 ± 0.45	0.32 ± 0.65	**< 0.001**
Unpleasant dreams	49 (7.87%)	114 (18.30%)	**< 0.001**	0.11 ± 0.40	0.26 ± 0.60	**< 0.001**
Avoid thoughts	48 (7.70%)	118 (18.94%)	**< 0.001**	0.11 ± 0.40	0.25 ± 0.59	**< 0.001**
**Sleep**	301 (48.31%)	433 (69.50%)	**< 0.001**	0.63 ± 0.76	1.13 ± 0.97	**< 0.001**
Functional disabilities						
Communication	124 (19.90%)	276 (44.30%)	**< 0.001**	0.24 ± 0.52	0.60 ± 0.78	**< 0.001**
Mobility	81 (13.00%)	147 (23.60%)	**< 0.001**	0.17 ± 0.50	0.34 ± 0.68	**< 0.001**
Personal care	20 (3.21%)	67 (10.75%)	**< 0.001**	0.04 ± 0.27	0.14 ± 0.45	**< 0.001**
Daily living	67 (10.75%)	165 (26.48%)	**< 0.001**	0.14 ± 0.44	0.36 ± 0.68	**< 0.001**
Social role	50 (8.03%)	135 (21.67%)	**< 0.001**	0.11 ± 0.43	0.28 ± 0.59	**< 0.001**
**Overall health** ^**‡**^	NA	NA	NA	7.47 ± 1.79	6.40 ± 1.81	**< 0.001**

NA: Not applicable.

^*^Data were expressed in *n* (percentage).

^†^Data were expressed in means ± standard deviation.

^‡^Zero refers to the worst overall health, and 10 refers to the best overall health.

### Other symptoms

As shown in Table 2, the mean number of the 25 predefined symptoms decreased from 3.61 ± 4.88 before service utilisation to 2.75 ± 4.79 immediately after service utilisation, and further to 2.30 ± 4.82 three months after service utilisation (*p* < 0.001). The presence of these symptoms is presented in Supplementary Table S1. Generally, the most present symptoms before service utilisation remained the most present after service utilisation and three months later, though with a decreasing trend in presence. The number of participants reporting various numbers of these symptoms is detailed in Supplementary Table S2. More than half (50.24%) of the participants reported having one or more of these 25 symptoms before service utilisation. It decreased to 34.51% after service utilisation and to 26.00% three months after service utilisation. Despite the overall trend of decreasing symptoms reported, the number of participants reporting more than 15 symptoms increased three months after service utilisation.

### Logistic regression analysis

The logistic regression analyses shed light on the factors influencing recovery from PCC. Supplementary Table S3 presents the results from the univariate logistic regression model for achieving an MID of symptoms three months after service utilisation. Supplementary Table S4 provides similar details for functional disabilities, the number of other symptoms, and overall health. We found that age group, female, full-time employment, the number of chronic diseases, and allied health services usage could be robust predictors of achieving all MIDs in symptoms. Similar results were observed between pre-infection and pre-service severity scores and the achievement of MIDs in symptoms, functional disabilities, and the number of other symptoms.

Supplementary Table S5 presents the full results of the multivariate logistic regression analyses, while Table 4 highlights the predictors that showed significant associations. The analyses revealed that pre-service severity score was a consistent predictor of recovery across various symptoms and functional disabilities, with aORs ranging from 4.23 to 133.43. This indicates that participants with more severe PCC were more likely to experience clinical improvement three months after service utilisation. Similarly, a negative association was observed between pre- and post-service overall health scores (aOR: 0.53; 95% CI: 0.46–0.60; *p* < 0.001), indicating that poorer self-reported pre-service health status was associated with increased odds of significant post-service improvement. Older age was also negatively associated with improvements in cough, throat sensitivity or voice changes, pain or discomfort, cognition, palpitations or dizziness, sleep, and communication, with aORs ranging from 0.71 to 0.84. Other predictors for recovery of symptoms, functional disabilities, and/or overall health included full-time employment, smoking, use of allied health services, pre-infection severity scores, and the number of chronic diseases, COVID-19 infections, and vaccine doses received.

**Table 4:** Predictors for achieving minimally important differences in symptoms, functional disabilities, and overall health three months after service utilisation: Multivariate logistic regressions.

Predictor	aOR (95% CI)	*P*-value
Breathlessness		
Full-time employment	2.57 (1.58, 4.18)	< 0.001
Severity score before service	4.96 (3.72, 6.59)	< 0.001
Smoking	0.34 (0.18, 0.65)	0.001
Cough/Throat sensitivity/Voice change		
Older in age	0.78 (0.69, 0.89)	< 0.001
Severity score before service	4.72 (3.62, 6.16)	< 0.001
Fatigue		
Severity score before service	4.46 (3.40, 5.87)	< 0.001
Smell/Taste		
Severity score before service	18.85 (11.60, 30.64)	< 0.001
Pain/Discomfort		
Older in age	0.81 (0.70, 0.94)	0.007
Number of chronic disease(s)	0.81 (0.66, 0.99)	0.043
Severity score before infection	0.74 (0.56, 0.99)	0.04
Severity score before service	4.44 (3.36, 5.86)	< 0.001
Cognition		
Older in age	0.84 (0.73, 0.98)	0.025
Severity score before service	4.25 (3.29, 5.50)	< 0.001
Palpitations/Dizziness		
Older in age	0.71 (0.61, 0.84)	< 0.001
Allied health services usage	1.08 (1.01, 1.17)	0.032
Severity score before service	8.67 (6.08, 12.38)	< 0.001
Post-exertional malaise		
Full-time employment	1.87 (1.15, 3.02)	0.011
Severity score before service	5.08 (3.87, 6.67)	< 0.001
Anxiety/Mood		
Severity score before service	4.98 (3.74, 6.64)	< 0.001
Sleep		
Older in age	0.81 (0.69, 0.96)	0.017
Full-time employment	1.71 (1.08, 2.69)	0.022
Severity score before infection	0.66 (0.50, 0.88)	0.004
Severity score before service	4.23 (3.27, 5.46)	< 0.001
Communication		
Older in age	0.72 (0.62, 0.85)	< 0.001
Number of COVID-19 infection(s)	0.32 (0.17, 0.59)	< 0.001
Severity score before service	7.81 (5.47, 11.15)	< 0.001
Mobility		
Number of COVID-19 vaccine dose(s)	1.72 (1.22, 2.44)	0.002
Severity score before infection	0.55 (0.31, 1.00)	0.049
Severity score before service	23.53 (13.39, 41.34)	< 0.001
Personal care		
Severity score before service	133.43 (39.53, 450.37)	< 0.001
Daily living		
Severity score before service	15.73 (10.05, 24.61)	< 0.001
Social role		
Severity score before service	25.53 (13.68, 46.51)	< 0.001
Overall health		
Older in age	0.81 (0.69, 0.95)	0.01
Allied health services usage	1.09 (1.02, 1.17)	0.009
Number of COVID-19 vaccine dose(s)	1.27 (1.05, 1.54)	0.012
Overall health score before service	0.53 (0.46, 0.60)	< 0.001

Note: Only statistically significant results (p-value < 0.05) are presented.

aOR: Adjusted odds ratio; CI: Confidence interval.

## Discussion

### Summary of results

A total of 623 participants from the PCC community-based rehabilitation support programme in Hong Kong completed all assessments in this longitudinal study. The results indicated that participants experienced statistically significant improvements in 16 out of the 17 domains (excluding personal care) concerning symptom severity, functional disability, and overall health status, as assessed by the Cantonese C19-YRSm, both immediately and three months post-service utilisation. Nevertheless, participants did not fully revert to their self-reported pre-COVID-19 health status. Multivariate logistic regression analyses revealed that younger participants and those with more severe symptoms prior to service were more likely to exhibit substantial clinical improvements across multiple domains of the questionnaire three months after service utilisation.

### Comparisons to similar studies

A range of multidisciplinary rehabilitation programmes for PCC have been developed and implemented globally, with subsequent evaluation studies conducted to assess their outcomes. For example, the PCR SIRIO 8 programme in Poland provided 97 individuals with PCC, who exhibited mild functional limitations, decreased muscle strength, or mild dyspnoea, with a personalised outpatient multidisciplinary rehabilitation package^22,23^. Following six weeks of individual and group interventions, the evaluation demonstrated significant improvements in participants’ body composition, dyspnoea, exercise tolerance, and physical fitness^23^. Similarly, in Spain, an 8-week multidisciplinary outpatient rehabilitation programme was tailored for 43 individuals presenting with neurological, cognitive, and musculoskeletal symptoms of PCC^24^. The evaluation revealed notable improvements in motor functional independence, upper and lower limb functionality, the impact of fatigue on daily activities, respiratory muscle strength, cognitive performance, and quality of life^24^. Together with our findings, these results highlight the potential of person-centred, community-based, multidisciplinary approaches to the rehabilitation journey of individuals with PCC. However, the specific interventions included in such programmes will inevitably vary depending on the healthcare systems and resources available within different countries and communities.

### Implications for practice

Our programme integrated all major health and allied health services across Hong Kong to provide co-designed, coordinated care to participants. In particular, BMDs and nurses were responsible for conducting general medical assessments and ongoing symptom monitoring, alongside providing health education on symptom management. Physiotherapists and occupational therapists focused on physical rehabilitation and functional recovery, while dietitians offered tailored nutritional support to promote overall health and recovery.

A distinctive feature was the inclusion of TCM, a well-established healthcare modality alongside conventional medicine, which plays a significant role in managing chronic diseases in outpatient settings across the territory^25^. Participants referred to TCM services by NGO case managers (i.e., social workers) received consultations and treatments at private TCM clinics within their communities. These treatments included herbal medicine, acupuncture, massage, or a combination of therapies, depending on the clinical judgement of practitioners. Recent reviews have highlighted the effectiveness of acupuncture in alleviating neurological and neuropsychiatric symptoms among individuals with PCC^26^, as well as the potential of specific herbal prescriptions in reducing PCC-related chronic fatigue^27^. Other existing research, although not specifically targeting PCC, may also contribute evidence on managing chronic gastrointestinal symptoms through herbal medicine^28–30^. In light of the positive outcomes observed in this study and the growing evidence supporting the benefits of acupuncture and herbal medicine for PCC-related symptoms, future community-based multidisciplinary care models may consider integrating TCM or other complementary and alternative medical services. This is particularly relevant in regions where these modalities are accepted, accessible, and affordable.

Future community-based multidisciplinary care models may benefit from incorporating self-management and supported self-management, which are increasingly recognised as important elements of PCC rehabilitation^31,32^. These approaches have the potential to contribute to symptom management, quality of life improvements, and the restoration of productivity among those affected. As with multidisciplinary rehabilitation plans, self-management and supported self-management plans are recommended to be co-designed and agreed upon by the self-management facilitator, the person with PCC, and his/her family or carers, following the principle of shared decision-making^32^. Additional self-management resources, including those published by the World Health Organization^33^, may serve as useful adjuncts to professional healthcare support.

### Implications for research

Our findings revealed that, even after completing the rehabilitation programme for up to 12 weeks, participants were unable to fully return to their self-reported pre-COVID-19 health status. To determine whether this outcome is due to the duration of the programme or the persistent detrimental effects of COVID-19 infection, a longer-term programme is warranted to help assess the long-term effectiveness of the multidisciplinary approach and provide a clearer understanding of its impact on recovery. The observed association between younger age and greater improvement in PCC aligns with findings from international cohort studies^34–36^. Age-related impairments in immune regulation, including reduced control of inflammatory processes, diminished T‑cell function, and lower production of naïve immune cells, are associated with delayed recovery and poorer outcomes following COVID-19 infection^37^. These differences in immunological responsiveness and physiological resilience may be among the factors contributing to the greater recovery observed in younger individuals during post-acute COVID-19 rehabilitation. However, the observed negative relationship between initial symptom severity and PCC recovery rate contrasts with findings from existing studies^38^. This discrepancy may reflect variations in healthcare engagement, motivation to adhere to rehabilitation protocols, or the timing and intensity of multidisciplinary interventions. Identifying these predictors could inform targeted, personalised rehabilitation strategies, while future research should explore the mechanisms underlying these associations and their interaction with specific therapeutic interventions to optimise recovery outcomes. Incorporating waitlist control groups for various health and allied health services in future programmes could also help distinguish clinical improvements attributable to the intervention, thereby guiding evidence-based resource allocation.

Given the considerable resources required to deliver multidisciplinary rehabilitation programmes, including personnel, facilities, and materials, it is important to assess not only their clinical effectiveness but also their economic feasibility. Evaluating the costs relative to the health benefits achieved would provide valuable insight into the value of such interventions and inform decisions about their broader implementation. In addition, examining potential strategies for optimising efficiency, such as task-sharing among healthcare professionals, prioritising high-need participants, or integrating digital components, could help enhance scalability without compromising quality of care. Future research incorporating formal cost-effectiveness analyses alongside clinical outcomes would enable policymakers and service providers to make evidence-based decisions regarding resource allocation and the potential expansion of such programmes to larger populations.

Future studies should implement procedures to ensure the collection of a minimum de-identified dataset for all recruited participants, including those who do not complete follow-up assessments. Collecting basic participant characteristics and assigning unique study identifiers at recruitment would allow linkage between recruitment and survey data while preserving confidentiality, support clearer reporting of participant flow, and enable comparisons between participants with complete and incomplete follow-up data.

### Limitations

This longitudinal study has several limitations. First, in the C19-YRSm, the participants were asked to recall the severity of their symptoms and functional impairments prior to COVID-19 infection, which could introduce recall bias, potentially leading to either an overestimation or underestimation of pre- and post-intervention differences^39^. Data on hospitalisation or intensive care during the acute infection phase were not collected. However, participants’ self-reported infection severity was considered a proxy for the intensity of their initial COVID-19 illness, encompassing those managed as outpatients as well as individuals who may have required hospital or intensive care. Second, despite the provision of cash incentives and flexible options for survey completion—including telephone interviews, online questionnaires, or a combination of both—the study experienced a substantial drop-out rate (51.18%). Participants who did not complete follow-up assessments were contacted multiple times; however, many were unreachable or declined further participation, and reasons for non-completion were not systematically recorded. As outcome data were unavailable for these participants, subgroup or outcome-based analyses among drop-outs were not feasible, limiting our ability to assess whether attrition occurred non-randomly. If loss to follow-up disproportionately affected participants with specific characteristics or outcome trajectories (e.g., those experiencing greater or lesser improvements), the estimated intervention effects may be subject to attrition-related selection bias, potentially resulting in over- or underestimation of the true effects^40^. Third, we acknowledge the potential value of comparing the characteristics of participants who completed all follow-up surveys with those who did not. However, the participating NGOs were only required to provide the research team with fully completed survey sets and de-identified participant information, which limited our ability to conduct such analyses. Fourth, due to privacy concerns and professional autonomy, we did not review participants’ treatment records, so we were unaware of the exact medical or health services received by each participant—only the frequency of consultations was recorded. As a result, we were unable to conduct further analyses comparing the effectiveness of TCM services, conventional medicine services, and allied health services. Fifth, the absence of a control group, which would have included participants receiving no services, limits our ability to determine whether the observed clinical improvements were solely attributable to the programme, especially since some PCC symptoms tend to improve over time^41^. Future studies comparing outcomes with the natural progression of the condition, as measured by the C19-YRS, are warranted. Finally, given the exploratory nature of this study and the large number of outcomes examined, there is a risk of inflated type I error^42^. As no formal adjustments for multiple comparisons were made, the findings should be interpreted with caution.

## Conclusions

Following participation in our PCC community-based rehabilitation support programme for up to 12 weeks, individuals with PCC demonstrated statistically significant improvements across most C19-YRSm domains, including symptom severity, functional disability, and overall health status, both immediately and three months post-programme completion. Despite these improvements, participants did not fully return to their self-reported pre-COVID-19 health status.

## Supplementary Information

Below is the link to the electronic supplementary material.

Supplementary Material 1

## Data Availability

All data supporting the findings of this study are available within the paper and its Supplementary Information.
