# Supplementary material for: A longitudinal evaluation of the community-based rehabilitation support programme for Post-COVID-19 condition in Hong Kong
Source: Sci Rep. 2026 Mar 31;16:10552. doi: 10.1038/s41598-026-46888-x (PMC13039197; doi:10.1038/s41598-026-46888-x)
Supplement: Supplementary file 1 — Supplementary Material 1 [file 41598_2026_46888_MOESM1_ESM.docx]

**A longitudinal evaluation of the community-based rehabilitation support programme for Post-COVID-19 Condition in Hong Kong**

**Supplementary file**

[Table S1. Prevalence of other symptoms before service utilisation, after service utilisation, and three months after service utilisation](#_Toc198738613)

[Table S2. Number of other symptoms before service utilisation, after service utilisation, and three months after service utilisation](#_Toc198738614)

[Table S3. Univariate logistic regression analyses of factors associated with achieving minimally important differences in symptoms three months after service utilisation](#_Toc198738615)

[Table S4. Univariate logistic regression analyses of factors associated with achieving minimally important differences in functional disabilities, number of other symptoms, and overall health three months after service utilisation](#_Toc198738616)

[Table S5. Multivariate logistic regression analyses of predictors for achieving minimally important differences in symptoms, functional disability, and overall health three months after service utilisation](#_Toc198738617)

# Table S1. Prevalence of other symptoms before service utilisation, after service utilisation, and three months after service utilisation

| **Other symptoms (in descending order at Baseline)** | **Prevalence (%)** | | |
| --- | --- | --- | --- |
|  | **Before service** | **After service** | **3 months after service** |
| Dry mouth/Mouth ulcers | 28.89 | 19.42 | 15.41 |
| Skin sensation | 26.97 | 20.55 | 15.57 |
| Visual changes | 24.56 | 18.30 | 16.21 |
| Hair loss | 23.92 | 18.62 | 15.25 |
| Weakness/Movement problems/Coordination problems | 20.87 | 17.50 | 11.56 |
| Bladder frequency, urgency, or incontinence | 20.87 | 16.05 | 14.45 |
| Change in appetite | 20.22 | 12.20 | 8.67 |
| Dry eyes/Redness of eyes | 18.94 | 14.61 | 12.20 |
| Acid reflux/Heartburn | 17.34 | 13.00 | 11.56 |
| Constipation, diarrhoea, or bowel incontinence | 17.34 | 12.52 | 10.43 |
| Tinnitus | 17.17 | 13.32 | 12.36 |
| Balance problems or falls | 15.57 | 12.84 | 11.08 |
| Fever | 13.64 | 8.83 | 7.54 |
| Nausea | 12.52 | 6.90 | 6.26 |
| Unintentional weight loss | 11.56 | 7.54 | 6.10 |
| Skin rash/Discolouration of skin | 11.40 | 11.24 | 8.67 |
| Swelling of feet or hands | 10.11 | 8.51 | 7.87 |
| Difficulty swallowing solids | 8.83 | 7.54 | 7.54 |
| Unintentional weight gain | 8.83 | 7.54 | 6.26 |
| Change in menstrual cycles of flow | 8.35 | 6.26 | 4.01 |
| Easy bruising or bleeding | 7.54 | 5.62 | 6.10 |
| Sleep apnoea | 6.26 | 4.65 | 3.21 |
| Difficulty swallowing liquids | 6.42 | 5.46 | 5.46 |
| New allergy | 5.30 | 4.49 | 4.33 |
| Thoughts about harming yourself | 2.73 | 1.77 | 2.09 |

# Table S2. Number of other symptoms before service utilisation, after service utilisation, and three months after service utilisation

| **Number of other symptoms** | **Before service** | **After service** | **3 months after service** |
| --- | --- | --- | --- |
| 0 | 310 (49.76%) | 408 (65.49%) | 461 (74.00%) |
| 1 | 24 (3.85%) | 13 (2.09%) | 11 (1.77%) |
| 2 | 27 (4.33%) | 15 (2.41%) | 16 (2.57%) |
| 3 | 37 (5.94%) | 15 (2.41%) | 7 (1.12%) |
| 4 | 19 (3.05%) | 19 (3.05%) | 7 (1.12%) |
| 5 | 26 (4.17%) | 15 (2.41%) | 9 (1.44%) |
| 6 | 25 (4.01%) | 22 (3.53%) | 11 (1.77%) |
| 7 | 26 (4.17%) | 14 (2.25%) | 17 (2.73%) |
| 8 | 25 (4.01%) | 15 (2.41%) | 10 (1.61%) |
| 9 | 17 (2.73%) | 17 (2.73%) | 11 (1.77%) |
| 10 | 18 (2.89%) | 14 (2.25%) | 5 (0.80%) |
| 11 | 8 (1.28%) | 6 (0.96%) | 11 (1.77%) |
| 12 | 12 (1.93%) | 8 (1.28%) | 4 (0.64%) |
| 13 | 9 (1.44%) | 11 (1.77%) | 5 (0.80%) |
| 14 | 12 (1.93%) | 4 (0.64%) | 4 (0.64%) |
| 15 or above | 28 (4.49%) | 27 (4.33%) | 34 (5.46%) |

*Note: Data expressed in n (percentage)*

# Table S3. Univariate logistic regression analyses of factors associated with achieving minimally important differences in symptoms three months after service utilisation

| **Variables** | **Breathless-ness** | **Cough / Throat sensitivity / Voice change** | **Fatigue** | **Smell / Taste** | **Pain / Discomfort** | **Cognition** | **Palpitations / Dizziness** | **Post-exertional malaise** | **Anxiety / Mood** | **Sleep** |
| --- | --- | --- | --- | --- | --- | --- | --- | --- | --- | --- |
| Age (continuous) | **0.85**  **(0.76, 0.94)** | **0.88**  **(0.79, 0.98)** | **0.79**  **(0.71, 0.88)** | 0.93  (0.83, 1.06) | **0.87**  **(0.78, 0.97)** | **0.85**  **(0.77, 0.95)** | **0.86**  **(0.77, 0.96)** | **0.89**  **(0.80, 0.99)** | 0.93  (0.83, 1.04) | **0.81**  **(0.73, 0.90)** |
|  | **0.003** | **0.017** | **<0.001** | 0.285 | **0.010** | **0.004** | **0.008** | **0.037** | 0.189 | **<0.001** |
| Female (biological sex) | 1.16  (0.82, 1.64) | 1.04  (0.73, 1.47) | 1.11  (0.79, 1.56) | 1.04  (0.70, 1.55) | **1.58**  **(1.11, 2.25)** | 1.28  (0.91, 1.81) | **1.45**  **(1.01, 2.07)** | 1.32  (0.94, 1.87) | 1.13  (0.79, 1.62) | 1.01  (0.72, 1.42) |
|  | 0.400 | 0.828 | 0.535 | 0.837 | **0.011** | 0.155 | **0.045** | 0.110 | 0.507 | 0.964 |
| Body mass index | 1.01  (0.97, 1.05) | 1.01  (0.97, 1.05) | 1.00  (0.96, 1.04) | 1.01  (0.97, 1.06) | 0.97  (0.94, 1.02) | 1.02  (0.98, 1.06) | 0.98  (0.94, 1.02) | 1.02  (0.97, 1.06) | 1.00  (0.96, 1.05) | 0.98  (0.94, 1.02) |
|  | 0.750 | 0.643 | 0.921 | 0.561 | 0.233 | 0.319 | 0.280 | 0.464 | 0.921 | 0.395 |
| Smoking | **0.48**  **(0.28, 0.81)** | 0.89  (0.55, 1.45) | 1.09  (0.68, 1.75) | 1.22  (0.72, 2.07) | 0.80  (0.50, 1.30) | 0.70  (0.43, 1.14) | 1.01  (0.62, 1.64) | 0.70  (0.43, 1.14) | 1.40  (0.87, 2.27) | 1.22  (0.76, 1.95) |
|  | **0.006** | 0.652 | 0.715 | 0.466 | 0.377 | 0.153 | 0.956 | 0.153 | 0.165 | 0.409 |
| Full-time employment before COVID-19 infection | **1.82**  **(1.32, 2.52)** | 1.22  (0.88, 1.69) | **1.50**  **(1.09, 2.06)** | 1.11  (0.76, 1.60) | 1.24  (0.90, 1.71) | 1.31  (0.95, 1.80) | 1.04  (0.75, 1.44) | 1.35  (0.98, 1.85) | 1.01  (0.72, 1.40) | **1.59**  **(1.16, 2.19)** |
|  | **<0.001** | 0.227 | **0.013** | 0.594 | 0.181 | 0.095 | 0.823 | 0.067 | 0.970 | **0.004** |
| Number of COVID-19 infection(s) | 0.84  (0.57, 1.23) | 0.84  (0.57, 1.24) | 0.79  (0.55, 1.15) | 0.95  (0.62, 1.47) | 1.36  (0.94, 1.97) | 1.14  (0.79, 1.65) | 1.07  (0.73, 1.57) | 1.10  (0.76, 1.60) | 0.91  (0.61, 1.35) | 1.05  (0.72, 1.52) |
|  | 0.376 | 0.381 | 0.224 | 0.827 | 0.105 | 0.486 | 0.719 | 0.612 | 0.641 | 0.803 |
| Number of COVID-19 vaccine dose(s) | 1.10  (0.93, 1.30) | 1.07  (0.90, 1.27) | 1.12  (0.95, 1.32) | 1.04  (0.85, 1.26) | 1.11  (0.94, 1.32) | 1.07  (0.91, 1.27) | 1.03  (0.87, 1.23) | 1.07  (0.91, 1.27) | 0.97  (0.82, 1.15) | 1.13  (0.95, 1.34) |
|  | 0.281 | 0.433 | 0.180 | 0.702 | 0.226 | 0.426 | 0.699 | 0.426 | 0.733 | 0.161 |
| Number of chronic disease(s) | 0.87  (0.75, 1.01) | 0.91  (0.78, 1.04) | **0.84**  **(0.73, 0.97)** | 0.91  (0.77, 1.07) | **0.83**  **(0.72, 0.96)** | 0.91  (0.79, 1.04) | 0.95  (0.82, 1.09) | 1.06  (0.92, 1.22) | 0.97  (0.83, 1.12) | 0.89  (0.77, 1.02) |
|  | 0.061 | 0.172 | **0.016** | 0.255 | **0.011** | 0.176 | 0.443 | 0.411 | 0.639 | 0.096 |
| Stroke history | **0.12**  **(0.02, 0.92)** | 0.94  (0.31, 2.92) | 0.57  (0.19, 1.77) | 0.56  (0.12, 2.57) | 0.24  (0.05, 1.11) | 1.44  (0.48, 4.34) | 1.46  (0.48, 4.39) | 1.99  (0.64, 6.15) | 1.66  (0.55, 4.99) | 0.55  (0.17, 1.80) |
|  | **0.041** | 0.920 | 0.335 | 0.594 | 0.069 | 0.517 | 0.504 | 0.233 | 0.370 | 0.322 |
| Severity score before infection | **1.33**  **(1.07, 1.66)** | 1.01  (0.81, 1.27) | **1.30**  **(1.05, 1.61)** | **1.62**  **(1.21, 2.16)** | **1.37**  **(1.11, 1.69)** | **1.51**  **(1.20, 1.91)** | **2.01**  **(1.51, 2.68)** | **1.79**  **(1.38, 2.32)** | **2.10**  **(1.62, 2.72)** | **1.23**  **(1.00, 1.52)** |
|  | **0.011** | 0.902 | **0.016** | **0.001** | **0.004** | **<0.001** | **<0.001** | **<0.001** | **<0.001** | **0.049** |
| Severity score before service | **3.75**  **(2.95, 4.77)** | **4.43**  **(3.43, 5.72)** | **4.13**  **(3.20, 5.31)** | **17.52**  **(11.00, 27.89)** | **3.30**  **(2.64, 4.13)** | **3.88**  **(3.06, 4.92)** | **6.98**  **(5.13, 9.50)** | **4.58**  **(3.57, 5.87)** | **4.41**  **(3.44, 5.65)** | **3.11**  **(2.52, 3.83)** |
|  | **<0.001** | **<0.001** | **<0.001** | **<0.001** | **<0.001** | **<0.001** | **<0.001** | **<0.001** | **<0.001** | **<0.001** |
| Western medical services usage | 1.58  (0.98, 2.54) | 0.77  (0.47, 1.28) | 0.90  (0.57, 1.41) | 0.89  (0.51, 1.55) | 1.42  (0.89, 2.27) | 0.99  (0.63, 1.56) | 1.13  (0.71, 1.78) | 1.10  (0.70, 1.74) | 1.17  (0.74, 1.86) | 1.25  (0.79, 1.97) |
|  | 0.060 | 0.316 | 0.639 | 0.672 | 0.139 | 0.969 | 0.609 | 0.671 | 0.503 | 0.345 |
| Chinese medical services usage | 0.98  (0.93, 1.04) | 1.02  (0.96, 1.07) | 1.04  (0.98, 1.09) | **1.07**  **(1.00, 1.14)** | 0.99  (0.93, 1.04) | 0.97  (0.92, 1.03) | 1.06  (1.00, 1.12) | 1.05  (1.00, 1.11) | 1.06  (1.00, 1.12) | 0.99  (0.94, 1.05) |
|  | 0.528 | 0.573 | 0.205 | **0.049** | 0.644 | 0.305 | 0.063 | 0.063 | 0.058 | 0.711 |
| Allied health services usage | 1.06  (1.00, 1.12) | 1.03  (0.97, 1.09) | 0.97  (0.92, 1.03) | 0.96  (0.89, 1.03) | **1.07**  **(1.01, 1.14)** | 0.96  (0.91, 1.02) | **1.06**  **(1.01, 1.13)** | 1.00  (0.95, 1.06) | 0.96  (0.91, 1.03) | 1.04  (0.98, 1.10) |
|  | 0.053 | 0.352 | 0.301 | 0.240 | **0.018** | 0.212 | **0.032** | 0.877 | 0.250 | 0.189 |

*Note: Data expressed in odds ratios (95% confidence intervals) and p-values.*

# Table S4. Univariate logistic regression analyses of factors associated with achieving minimally important differences in functional disabilities, number of other symptoms, and overall health three months after service utilisation

| **Variables** | **Communication** | **Mobility** | **Personal care** | **Daily living** | **Social role** | **Number of other symptoms** | **Overall health** |
| --- | --- | --- | --- | --- | --- | --- | --- |
| Age (continuous) | **0.89 (0.79, 1.00)** | **1.21 (1.06, 1.39)** | **1.63 (1.25, 2.13)** | 1.04 (0.92, 1.18) | 1.14 (0.99, 1.31) | 1.03 (0.92, 1.16) | **0.80 (0.71, 0.89)** |
|  | **0.049** | **0.004** | **<0.001** | 0.510 | 0.074 | 0.629 | **<0.001** |
| Female (biological sex) | 1.38 (0.95, 2.01) | **1.68 (1.08, 2.62)** | **4.09 (1.43, 11.69)** | **1.59 (1.06, 2.41)** | 1.60 (0.98, 2.59) | 1.09 (0.74, 1.58) | 1.11 (0.79, 1.55) |
|  | 0.093 | **0.021** | **0.009** | **0.027** | 0.058 | 0.672 | 0.564 |
| Body mass index | 1.00 (0.96, 1.04) | **1.06 (1.01, 1.11)** | 1.03 (0.95, 1.11) | 1.03 (0.98, 1.08) | **1.06 (1.01, 1.12)** | 0.97 (0.93, 1.02) | 0.99 (0.95, 1.03) |
|  | 0.927 | **0.018** | 0.511 | 0.241 | **0.023** | 0.210 | 0.744 |
| Smoking | 0.86 (0.51, 1.45) | 1.06 (0.60, 1.86) | 0.79 (0.27, 2.28) | 0.84 (0.48, 1.46) | 1.13 (0.62, 2.07) | 1.25 (0.75, 2.06) | 0.75 (0.47, 1.20) |
|  | 0.576 | 0.847 | 0.661 | 0.529 | 0.689 | 0.391 | 0.235 |
| Full-time employment before COVID-19 infection | 1.17 (0.83, 1.64) | **0.42 (0.28, 0.64)** | **0.18 (0.07, 0.47)** | 0.78 (0.54, 1.12) | **0.51 (0.33, 0.80)** | 1.04 (0.73, 1.48) | **1.41 (1.03, 1.94)** |
|  | 0.374 | **<0.001** | **<0.001** | 0.180 | **0.003** | 0.814 | **0.033** |
| Number of COVID-19 infection(s) | **0.57 (0.36, 0.88)** | 0.92 (0.58, 1.45) | 0.70 (0.29, 1.67) | 1.18 (0.78, 1.78) | 1.02 (0.63, 1.66) | 0.78 (0.50, 1.19) | 0.91 (0.63, 1.32) |
|  | **0.012** | 0.713 | 0.420 | 0.445 | 0.934 | 0.245 | 0.625 |
| Number of COVID-19 vaccine dose(s) | 1.07 (0.89, 1.28) | **1.34 (1.07, 1.69)** | 1.07 (0.75, 1.53) | 1.07 (0.88, 1.30) | 1.14 (0.91, 1.44) | 0.95 (0.79, 1.14) | **1.20 (1.02, 1.43)** |
|  | 0.469 | **0.010** | 0.718 | 0.501 | 0.258 | 0.606 | **0.030** |
| Number of chronic disease(s) | 0.91 (0.78, 1.06) | **1.24 (1.06, 1.45)** | **1.31 (1.02, 1.67)** | 1.02 (0.87, 1.19) | 1.06 (0.89, 1.27) | 1.07 (0.92, 1.24) | **0.81 (0.70, 0.93)** |
|  | 0.226 | **0.006** | **0.033** | 0.804 | 0.498 | 0.405 | **0.003** |
| Stroke history | 1.89 (0.63, 5.71) | 1.63 (0.49, 5.36) | 2.90 (0.62, 13.57) | 1.87 (0.60, 5.79) | 2.19 (0.66, 7.24) | 1.59 (0.51, 4.92) | 0.59 (0.19, 1.82) |
|  | 0.256 | 0.425 | 0.177 | 0.280 | 0.200 | 0.423 | 0.358 |
| Severity score before infection | **3.55 (2.48, 5.08)** | **3.12 (2.15, 4.52)** | **4.29 (2.19, 8.41)** | **2.93 (1.93, 4.43)** | **3.69 (2.33, 5.85)** | NA | NA |
|  | **<0.001** | **<0.001** | **<0.001** | **<0.001** | **<0.001** | NA | NA |
| Severity score before service | **6.72 (4.96, 9.12)** | **14.88 (9.47, 23.40)** | **83.08 (29.57, 233.39)** | **13.62 (9.09, 20.42)** | **20.68 (12.09, 35.36)** | NA | NA |
|  | **<0.001** | **<0.001** | **<0.001** | **<0.001** | **<0.001** | NA | NA |
| Number of other symptoms before service | NA | NA | NA | NA | NA | 0.99 (0.96, 1.03) | NA |
|  | NA | NA | NA | NA | NA | 0.706 | NA |
| Overall health score before infection | NA | NA | NA | NA | NA | NA | 0.93 (0.85, 1.01) |
|  | NA | NA | NA | NA | NA | NA | 0.094 |
| Overall health score before service | NA | NA | NA | NA | NA | NA | **0.58 (0.51, 0.65)** |
|  | NA | NA | NA | NA | NA | NA | **<0.001** |
| Western medical services usage | **1.71 (1.07, 2.76)** | **1.78 (1.10, 2.90)** | 1.13 (0.48, 2.67) | 1.20 (0.74, 1.96) | 1.61 (0.98, 2.67) | 1.21 (0.75, 1.94) | 1.27 (0.79, 2.02) |
|  | **0.026** | **0.019** | 0.778 | 0.460 | 0.062 | 0.436 | 0.322 |
| Chinese medical services usage | 0.98 (0.93, 1.04) | 1.01 (0.95, 1.08) | 1.07 (0.94, 1.21) | 0.99 (0.93, 1.05) | 0.95 (0.89, 1.02) | 1.02 (0.96, 1.08) | 0.98 (0.93, 1.04) |
|  | 0.526 | 0.680 | 0.297 | 0.652 | 0.200 | 0.516 | 0.587 |
| Allied health services usage | 1.01 (0.95, 1.07) | 1.00 (0.94, 1.07) | 0.97 (0.86, 1.11) | 1.04 (0.98, 1.10) | 1.03 (0.96, 1.10) | 1.01 (0.95, 1.07) | **1.07 (1.00, 1.13)** |
|  | 0.829 | 0.922 | 0.689 | 0.249 | 0.426 | 0.750 | **0.036** |

*Note: Data expressed in odds ratios (95% confidence intervals) and p-values.*

NA: Not applicable.

# Table S5. Multivariate logistic regression analyses of predictors for achieving minimally important differences in symptoms, functional disability, and overall health three months after service utilisation

| **Predictor** | **aOR (95% CI)** | | ***P*-value** | |
| --- | --- | --- | --- | --- |
| **Breathlessness** | | | |  |
| Older in age | 0.93 (0.79, 1.11) | | 0.438 | |
| Allied health services usage | 1.06 (0.99, 1.13) | | 0.109 | |
| **Full-time employment before COVID-19 infection** | **2.57 (1.58, 4.18)** | | **<0.001** | |
| Number of chronic disease(s) | 0.90 (0.73, 1.11) | | 0.323 | |
| Severity score before infection | 0.81 (0.60, 1.09) | | 0.160 | |
| **Severity score before service** | **4.96 (3.72, 6.59)** | | **<0.001** | |
| **Smoking** | **0.34 (0.18, 0.65)** | | **0.001** | |
| Stroke history | 0.18 (0.02, 1.79) | | 0.144 | |
| Western medical services usage | 1.13 (0.66, 1.94) | | 0.649 | |
| **Cough / Throat sensitivity / Voice change** | | | |  |
| **Older in age** | **0.78 (0.69, 0.89)** | | **<0.001** | |
| **Severity score before service** | **4.72 (3.62, 6.16)** | | **<0.001** | |
| **Fatigue** | | | |  |
| Older in age | 0.92 (0.78, 1.09) | | 0.352 | |
| Full-time employment before COVID-19 infection | 1.29 (0.83, 2.02) | | 0.256 | |
| Number of chronic disease(s) | 0.84 (0.69, 1.02) | | 0.077 | |
| Severity score before infection | 0.84 (0.65, 1.08) | | 0.170 | |
| **Severity score before service** | **4.46 (3.40, 5.87)** | | **<0.001** | |
| **Smell / Taste** | | | |  |
| Severity score before infection | 0.74 (0.50, 1.11) | | 0.149 | |
| **Severity score before service** | **18.85 (11.60, 30.64)** | | **<0.001** | |
| Chinese medical services usage | 1.03 (0.93, 1.13) | | 0.616 | |
| **Pain / Discomfort** | | | |  |
| **Older in age** | **0.81 (0.70, 0.94)** | | **0.007** | |
| Allied health services usage | 1.06 (0.99, 1.13) | | 0.097 | |
| Female (biological sex) | 0.97 (0.64, 1.47) | | 0.880 | |
| **Number of chronic disease(s)** | **0.81 (0.66, 0.99)** | | **0.043** | |
| **Severity score before infection** | **0.74 (0.56, 0.99)** | | **0.040** | |
| **Severity score before service** | **4.44 (3.36, 5.86)** | | **<0.001** | |
| Stroke history | 0.20 (0.03, 1.15) | | 0.071 | |
| **Cognition** | | | |  |
| **Older in age** | **0.84 (0.73, 0.98)** | | **0.025** | |
| Full-time employment before COVID-19 infection | 1.32 (0.84, 2.06) | | 0.230 | |
| Severity score before infection | 0.86 (0.64, 1.14) | | 0.292 | |
| **Severity score before service** | **4.25 (3.29, 5.50)** | | **<0.001** | |
| **Palpitations / Dizziness** | | | |  |
| **Older in age** | **0.71 (0.61, 0.84)** | | **<0.001** | |
| **Allied health services usage** | **1.08 (1.01, 1.17)** | | **0.032** | |
| Female (biological sex) | 0.66 (0.41, 1.07) | | 0.090 | |
| Severity score before infection | 0.85 (0.58, 1.24) | | 0.390 | |
| **Severity score before service** | **8.67 (6.08, 12.38)** | | **<0.001** | |
| Chinese medical services usage | 1.01 (0.93, 1.09) | | 0.884 | |
| **Post-exertional malaise** | | | |  |
| Older in age | 0.94 (0.80, 1.11) | | 0.464 | |
| **Full-time employment before COVID-19 infection** | **1.87 (1.15, 3.02)** | | **0.011** | |
| Severity score before infection | 0.83 (0.61, 1.15) | | 0.263 | |
| **Severity score before service** | **5.08 (3.87, 6.67)** | | **<0.001** | |
| Chinese medical services usage | 1.06 (0.99, 1.14) | | 0.115 | |
| **Anxiety / Mood** | | | |  |
| Severity score before infection | 0.73 (0.52, 1.02) | | 0.062 | |
| **Severity score before service** | **4.98 (3.74, 6.64)** | | **<0.001** | |
| Chinese medical services usage | 1.02 (0.95, 1.10) | | 0.513 | |
| **Sleep** | | | |  |
| **Older in age** | **0.81 (0.69, 0.96)** | | **0.017** | |
| **Full-time employment before COVID-19 infection** | **1.71 (1.08, 2.69)** | | **0.022** | |
| Number of chronic disease(s) | 0.93 (0.77, 1.12) | | 0.446 | |
| **Severity score before infection** | **0.66 (0.50, 0.88)** | | **0.004** | |
| **Severity score before service** | **4.23 (3.27, 5.46)** | | **<0.001** | |
| **Communication** | | | |  |
| **Older in age** | **0.72 (0.62, 0.85)** | **<0.001** | |  |
| Female (biological sex) | 0.96 (0.58, 1.58) | 0.873 | |  |
| **Number of COVID-19 infection(s)** | **0.32 (0.17, 0.59)** | **<0.001** | |  |
| Severity score before infection | 1.13 (0.72, 1.76) | 0.598 | |  |
| **Severity score before service** | **7.81 (5.47, 11.15)** | **<0.001** | |  |
| Western medical services usage | 1.78 (0.97, 3.28) | 0.063 | |  |
| **Mobility** | | | |  |
| Older in age | 0.82 (0.64, 1.06) | 0.126 | |  |
| Body Mass Index (BMI) | 1.01 (0.94, 1.08) | 0.777 | |  |
| Full-time employment before COVID-19 infection | 0.73 (0.37, 1.43) | 0.358 | |  |
| Female (biological sex) | 0.77 (0.40, 1.46) | 0.423 | |  |
| Number of chronic disease(s) | 0.79 (0.58, 1.08) | 0.138 | |  |
| **Number of COVID-19 vaccine dose(s)** | **1.72 (1.22, 2.44)** | **0.002** | |  |
| **Severity score before infection** | **0.55 (0.31, 1.00)** | **0.049** | |  |
| **Severity score before service** | **23.53 (13.39, 41.34)** | **<0.001** | |  |
| Western medical services usage | 1.15 (0.59, 2.22) | 0.684 | |  |
| **Personal care** | | | |  |
| Older in age | 1.25 (0.75, 2.08) | 0.396 | |  |
| Full-time employment before COVID-19 infection | 0.99 (0.22, 4.53) | 0.993 | |  |
| Female (biological sex) | 1.15 (0.28, 4.69) | 0.843 | |  |
| Number of chronic disease(s) | 0.54 (0.31, 0.93) | 0.028 | |  |
| Severity score before infection | 0.40 (0.12, 1.37) | 0.144 | |  |
| **Severity score before service** | **133.43 (39.53, 450.37)** | **<0.001** | |  |
| **Daily living** | | | |  |
| Female (biological sex) | 0.81 (0.45, 1.46) | 0.486 | |  |
| Severity score before infection | 0.60 (0.33, 1.08) | 0.090 | |  |
| **Severity score before service** | **15.73 (10.05, 24.61)** | **<0.001** | |  |
| **Social role** | | | |  |
| Older in age | 0.92 (0.71, 1.19) | 0.533 | |  |
| Body Mass Index (BMI) | 0.99 (0.91, 1.07) | 0.732 | |  |
| Full-time employment before COVID-19 infection | 1.41 (0.65, 3.08) | 0.384 | |  |
| Female (biological sex) | 0.83 (0.41, 1.67) | 0.594 | |  |
| Severity score before infection | 0.77 (0.40, 1.48) | 0.437 | |  |
| **Severity score before service** | **25.53 (13.68, 46.51)** | **<0.001** | |  |
| Western medical services usage | 0.91 (0.37, 2.23) | 0.841 | |  |
| **Overall health** | | | |  |
| **Older in age** | **0.81 (0.69, 0.95)** | **0.010** | |  |
| **Allied health services usage** | **1.09 (1.02, 1.17)** | **0.009** | |  |
| Full-time employment before COVID-19 infection | 1.29 (0.83, 1.98) | 0.254 | |  |
| Number of chronic disease(s) | 0.88 (0.73, 1.06) | 0.180 | |  |
| **Number of COVID-19 vaccine dose(s)** | **1.27 (1.05, 1.54)** | **0.012** | |  |
| Overall health score before infection | 1.07 (0.96, 1.19) | 0.221 | |  |
| **Overall health score before service** | **0.53 (0.46, 0.60)** | **<0.001** | |  |

aOR: Adjusted odds ratio; CI: Confidence interval
